# Bacterial community assemblages in classroom floor dust of 50 public schools in a large city: characterization using 16S rRNA sequences and associations with environmental factors

**DOI:** 10.1186/s40168-020-00954-2

**Published:** 2021-01-20

**Authors:** Ju-Hyeong Park, Angela R. Lemons, Jerry Roseman, Brett J. Green, Jean M. Cox-Ganser

**Affiliations:** 1 Respiratory Health Division, National Institute for Occupational Safety and Health, Morgantown, WV USA; 2 Philadelphia Federation of Teachers Health & Welfare Fund & Union, Philadelphia, PA USA

**Keywords:** School, Classroom, Bacteria, Microbiome, Moisture damage

## Abstract

Characterizing indoor microbial communities using molecular methods provides insight into bacterial assemblages present in environments that can influence occupants’ health. We conducted an environmental assessment as part of an epidemiologic study of 50 elementary schools in a large city in the northeastern USA. We vacuumed dust from the edges of the floor in 500 classrooms accounting for 499 processed dust aliquots for 16S Illumina MiSeq sequencing to characterize bacterial assemblages. DNA sequences were organized into operational taxonomic units (OTUs) and identified using a database derived from the National Center for Biotechnology Information. Bacterial diversity and ecological analyses were performed at the genus level. We identified 29 phyla, 57 classes, 148 orders, 320 families, 1193 genera, and 2045 species in 3073 OTUs. The number of genera per school ranged from 470 to 705. The phylum *Proteobacteria* was richest of all while *Firmicutes* was most abundant. The most abundant order included *Lactobacillales*, *Spirulinales*, and *Clostridiales*. *Halospirulina* was the most abundant genus, which has never been reported from any school studies before. Gram-negative bacteria were more abundant and richer (relative abundance = 0.53; 1632 OTUs) than gram-positive bacteria (0.47; 1441). Outdoor environment-associated genera were identified in greater abundance in the classrooms, in contrast to homes where human-associated bacteria are typically more abundant. Effects of school location, degree of water damage, building condition, number of students, air temperature and humidity, floor material, and classroom’s floor level on the bacterial richness or community composition were statistically significant but subtle, indicating relative stability of classroom microbiome from environmental stress. Our study indicates that classroom floor dust had a characteristic bacterial community that is different from typical house dust represented by more gram-positive and human-associated bacteria. Health implications of exposure to the microbiomes in classroom floor dust may be different from those in homes for school staff and students.

**Video abstract**

**Supplementary Information:**

The online version contains supplementary material available at 10.1186/s40168-020-00954-2.

## Background

Microbes are ubiquitous in the environment and there are at least the same order of bacteria as the number of cells (3.0 × 10^13^) in the human body [1]. The human microbiome constantly interacts with the microbiomes of the surrounding environments. Studies on the human microbiome have shown that most microbes native to our bodies are not pathogens but essential for functional human homeostasis [2, 3]. Still, the scientific community incompletely understands how environmental microbiomes interact with the human microbiome or affect human health. To understand this relationship, microbes in the environment and in the human body need to be fully characterized. The recent development of high-throughput sequencing technology has provided a powerful tool to analyze all genetic material in samples, and has led to the discovery of microbial taxa that had never been identified before using the traditional culture methods [4, 5].

Over the past decade, there has been much interest in how microbiomes in the built environments affect respiratory illnesses such as asthma. A few environmental microbiome studies involving children suggested that exposure to a richer bacterial microbiome in house dust might have a protective effect on the development of asthma, atopy, or wheezing [6–9]. These studies also documented certain bacterial taxa associated with the protective effects such as the order *Actinomycetales*; the families *Prevotellaceae*, *Lachnospiraceae*, and *Rukinococcaceae*; the genera *Clostridium*, *Facklamia*, *Acinetobacter*, *Lactobacillus*, *Jeotgalicoccus*, *Corynebacterium*, and *Neisseria*; and the species *Staphylococcus sciuri*. In contrast, a pilot randomized controlled trial of 25 children reported that an inverse Simpson index used to measure classroom bacterial diversity was significantly associated with increased odds of asthma symptoms, whereas home bacterial diversity was not [10]. Another study reported an adverse effect of home bacterial richness (the number of different bacterial taxa) on severity of asthma in children [11]. Altogether, available peer-reviewed studies suggest that the effects of environmental bacteria on human health are complex; not only in bacterial richness but community composition, and presence and abundance of specific taxa may also play important roles.

The environmental microbiome is one of the core components in human exposures in the built environments that significantly contribute to occupants’ health, and thus better understanding of its role in health and environments is crucial [12]. The committee of the 2017 National Academies’ report on “Microbiomes of the Built Environment” emphasized the need for fundamental research on surface microbiome sources in indoor environments [2]. In the USA, on average, school teachers spend more than 1950 h per year in schools whereas elementary school students spend approximately 1195 h per year [13, 14]. This demonstrates that school indoor environments contribute to environmental exposures more than any other locations for teachers and students except their home. Recently, the European SINPHONIE study reported that increased exposures to particulate matter and volatile organic compounds in school classrooms were associated with upper and lower airways, eye, and systemic disorders in school children [15]. However, health effects of exposures to classroom microbiomes in students and school staff are not well understood. Although there are a number of studies published about indoor microbiomes, most of them focused on residential environments or university lecture rooms [16–18]. Only a limited number of studies using high-throughput molecular methods have characterized classroom microbiomes in primary schools [10, 19]. In our study, we characterized the bacterial microbiome in floor dust of classrooms in 50 elementary schools in a large US city. We also examined how school and classroom environmental factors influenced bacterial diversity and community composition in floor dust collected from 500 classrooms.

## Methods

### Environmental study

An environmental assessment was conducted in June 2015 as part of a cross-sectional epidemiologic study to examine associations of microbial exposures with health in school staff. We selected 50 elementary schools in Philadelphia, PA, to collect floor dust samples from ten selected classrooms in each school, for a total of 500 samples. The samples were collected from the floor near the edges of the room, at the junction of the floor and walls, where dust accumulation was observed. We measured and marked areas at the floor-wall junction around the full perimeter of each room (a total of 12 ft^2^). We vacuumed for 8-min the area with a precleaned crevice tool on a L’il Hummer backpack vacuum sampler (100 ft^3^/min, 1.5 horsepower, ProTeam Inc., Boise, ID, USA) equipped with a polyethylene filter sock (Midwest Filtration Company, Fairfield, OH, USA). After the collected dust was sieved with a mesh (pore size: 250 μm), it was homogenized by rotating on a 360-degree rotary arm shaker at 65 r.p.m for 2 h and then partitioned into aliquots. After the sampling was completed, the relative humidity (RH) and temperature in classroom air were measured and recorded. Information on the average number of students for each sampled classroom was obtained from classroom teachers.

We collected dampness and mold information from all accessible rooms in the schools by visual assessment and evaluated mold odor using the Dampness and Mold Assessment Tool developed by the National Institute for Occupational Safety and Health (https://www.cdc.gov/niosh/docs/2019-114/). The average dampness and mold score was calculated using scores on water damage-related factors for all room components in each classroom. Information on the facility condition index [FCI = (cost of assessed deficiencies)/(replacement value)] for each school was obtained from a publicly available facility condition assessment report at https://www.philasd.org/capitalprograms/wp-content/uploads/sites/18/2017/06/2015-FCA-Final-Report-1.pdf.

### Genomic DNA extraction

Genomic DNA (gDNA) was extracted from 499 of 500 floor dust samples (one sample had no dust collected) and reagent blanks (*n* = 30) as negative controls using the Roche High Pure PCR Template Preparation Kit (Roche Applied Sciences, Penzberg, Germany) as previously described [4, 20]. Five milligrams of dust was suspended in 250 μL of the kit’s tissue lysis buffer and added to a 2-mL reinforced tube containing 300 mg of 212–300 μm glass beads (Sigma-Aldrich, St. Louis, MO). The tubes were then processed in a bead mill homogenizer at a rate of 4.5 m/s for 30 s. After two cycles of centrifugation at 20,000×*g* for 1 min, the lysis supernatant was placed in a sterile 1.5-mL microcentrifuge tube with 20 μL CelLytic B Cell Lysis reagent (Sigma) and incubated at 37 °C for 15 min. Roche binding buffer (200 μL) and proteinase K solution (40 μL) were then added followed by a 10-min incubation at 70 °C. The samples were then washed and eluted as recommended by the manufacturer (Roche). Aliquots (20 μL) were stored at − 80 °C until shipment to the vendor for analysis.

### Bacterial 16S amplification, sequencing, and taxonomic identification

Extracted gDNA samples were submitted to RTL Genomics (Lubbuck, TX) for Illumina Mi-Seq sequencing of the bacterial 16S rRNA genes. The samples were amplified using the 357wF (CCTACGGGNGGCWGCAG) and 806R (GGACTACHVGGGTWTCTAAT) primer pair and sequenced as previously described [21]. The resulting sequences were quality checked to remove sequences with failed reads and low-quality tags and sequences that were less than half the expected amplicon length. Paired sequences were merged using the PEAR Illumina paired-end read merger, trimmed using a RTL internal trimming algorithm, and clustered into operational taxonomic units (OTUs) using a 96% similarity threshold using a USEARCH clustering algorithm [22, 23]. OTUs were selected using the UPARSE OTU selection algorithm [24] and chimeras were checked using the UCHIME chimera detection software [25]. For taxonomic identification, representative OTU sequences were compared to a database maintained by RTL Genomics of high-quality sequences derived from the National Center for Biotechnology Information database using a USEARCH global search algorithm [26].

### Statistical analysis

Taxonomy data from the sequencing results were analyzed with R using statistical packages vegan, tidyverse, gridExtra, Hmisc, ggplot2, Mass, and broom [27]. School and classroom-based Shannon-Weaver diversity (zero or positive number; the higher is the more diverse) and Bray-Curtis dissimilarity (constrained between 0 and 1; the higher is the more dissimilar) indices were calculated at the genus level due to 690 unidentifiable OTUs at the species level. The Bray-Curtis dissimilarity index is a taxonomy-based metric that allows the detection of small changes in community composition among the levels within a variable [28]. The Pielou’s evenness index (constrained between 0 and 1; the higher is the more even) was also calculated. Hierarchical cluster analysis using Ward minimum variance method (minimizing within-cluster variance) with the Bray-Curtis index was performed to categorize 50 schools into four clusters (A through D) [29]. To compare the water damage scores among the clusters, we performed multiple comparisons using the Tukey honestly significant difference (HSD) test [30]. Because the diversity indicated by the Shannon-Weaver index or number of OTUs is influenced by sample size (i.e., groups with more samples show a higher diversity), rarefied genus accumulation curves normalized by the same number of DNA sequences were examined to compare richness among the levels within each environmental factor [31]. Pearson correlation coefficients were calculated among Shannon-Weaver index and other environmental variables.

We used analysis of similarity (ANOSIM) to compare the mean ranks of between- and within-group (level) Bray-Curtis indices of the environmental variable (lower index has a lower rank value) [32]. R statistic was calculated by [4 (*B* − *W*)/*N* (*N* − 1)], where *B* and *W* are the averages of the between-group and within-group ranks, respectively, and *N* is the number of samples. Permutational multivariate analysis of variance (PERMANOVA) modeling was performed to examine the adjusted relationships between community dissimilarity and environmental variables in full space without ordination [27]. The environmental variables included area of school, floor level, floor material, and quartiles of FCI scores, average water damage scores, number of students, air temperature, and air RH. Because ANOSIM and PERMANOVA are sensitive to unequal dispersion among the groups for unbalanced design, we examined homogeneity of dispersion among the levels in each environmental variable. The homogeneity of dispersion test indicated that the groups had similar dispersion for the categorical variables tested in the study (*P* values > 0.05), except for RH (*P* value = 0.04) [32].

We used nonmetric multidimensional scaling (NMDS) to graphically present high dimensional data into a low dimensional space (*k* = 3 in our analysis) in a way that the dissimilarity information between groups is reserved [33]. Ellipses were constructed to show a 95% confidence intervals (CI) fitted into the spatial ordination, using standard error in chi-square distribution with two degrees of freedom. The NMDS is the most robust method that can handle any non-linear responses such as genus abundance [34]. Stress value (*S*, a statistic of goodness of fit) for the model was calculated [the smaller *S* value is the more favorable (< 0.2 as a rule of thumb)] [35]. We also fitted a distance-decay model to Bray-Curtis dissimilarities using the power-law function with 1000 permutations to examine if dissimilarity increases with distance between the pairwise schools [36]. We considered *P* ≤ 0.05 as statistically significant and *P* ≤ 0.10 as marginally significant.

## Results

### Bacterial richness, abundance, and diversity

**Fig. 1:**
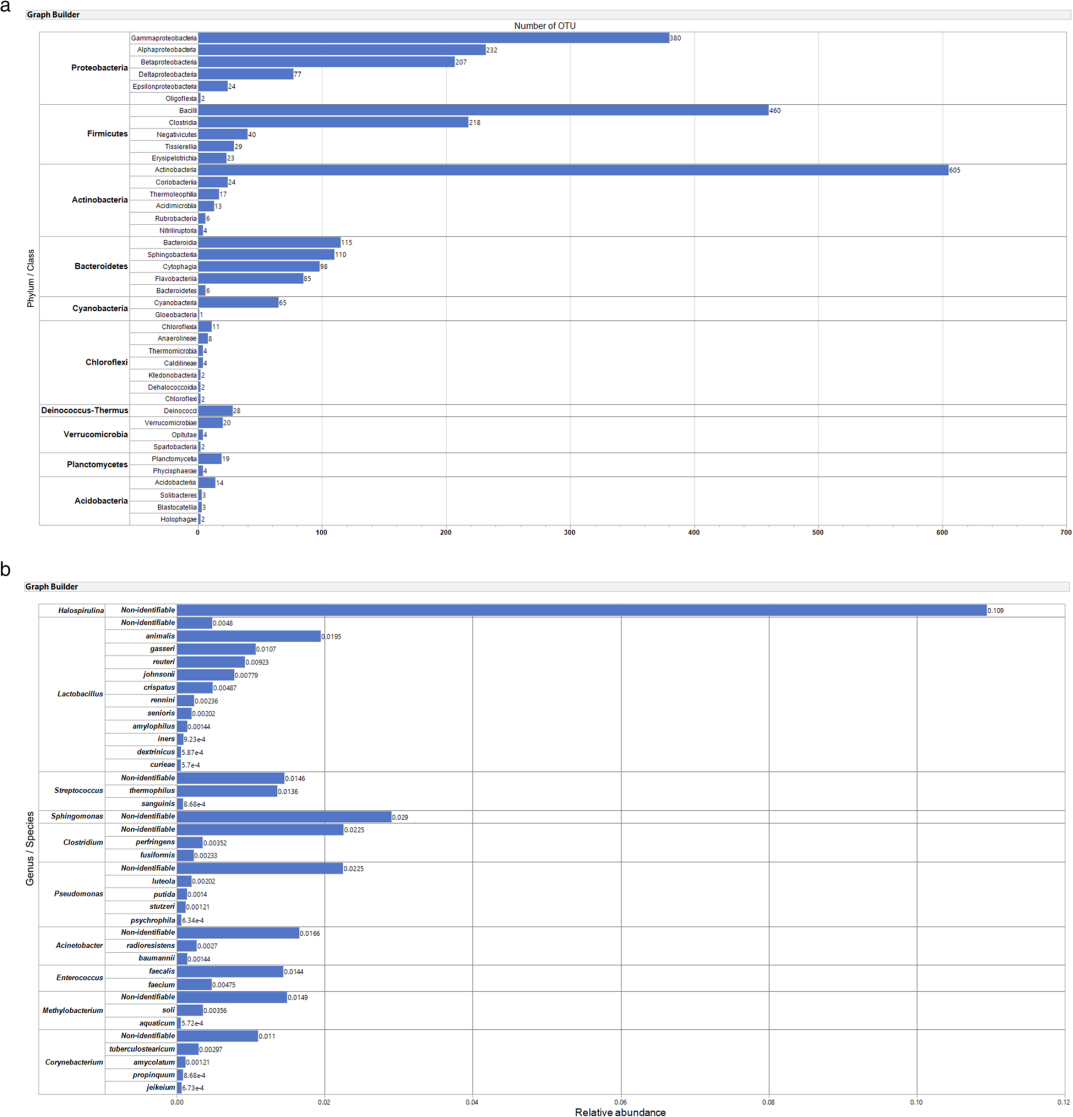
**a** Number of operational taxonomic units for each class of bacteria within the top 10 phyla. The bars are displayed by the descending order of the total number of OTUs in phylum and then in the class within each phylum. **b** Relative abundance of species within the top 10 most abundant genera. Because of too many OTUs, species that were smaller than 0.0005 in relative abundance are not presented. For *Halospirulina*, no species were identifiable; and for *Sphingomonas*, relative abundances of all four identified species were smaller than 0.0005 (thus, not in the figure). The bars are displayed by the descending order of relative abundance in genus and then in identified species within each genus

We identified 3073 unique OTUs from a total of 7.63 million sequences in the floor dust samples from the 499 classrooms, including 29 phyla, 57 classes, 148 orders, 320 families, 1193 genera, and 2045 species. Of the total 3073 OTUs, 1028 were not identifiable to the class or lower level. Among the 29 phyla, the *Proteobacteria* had the largest number of OTUs (922 identified OTUs at the class level), followed by *Firmicutes* (770), *Actinobacteria* (669), *Bacteroidetes* (414), and *Cyanobacteria* (66) (Fig. 1a). At the class level, *Actinobacteria* (the phylum *Actinobacteria*, 605 identified OTUs at the order level) was richest of all. However, the rank order of the top five richest phyla was not concordant with that of the top five most abundant phyla of which the phylum *Firmicutes* was most abundant (relative abundance: 0.29) (Supplemental Figure 1). The order *Lactobacillales* was most abundant (relative abundance: 0.14 in the phylum *Firmicutes*), followed by *Spirulinales* (0.11; *Cyanobacteria*), *Clostridiales* (0.07; *Firmicutes*), and *Bacteroidales* (0.07; *Bacteroidetes*). Of 1193 genera, *Halospirulina* was most abundant (the only genus within the order *Spirulinales*), followed by *Lactobacillus* (0.07) (Fig. 1b and Supplemental Figure 2). Of the most abundant top ten genera, only three [*Lactobacillus* (57 species identified), *Corynebacterium* (45), *Pseudomonas* (24)] were also included in the richest top ten genera. We identified 15 *Staphylococcus* species with 0.018 in relative abundance including unidentified species and five *Propionibacterium* species with small relative abundance (< 0.001). Gram-negative bacteria were more abundant (relative abundance = 0.54) and richer (1632 OTUs, 53%) than gram-positive bacteria [0.46; 1441 OTUs (47%), respectively] in the bacterial community of these schools.

**Fig. 2:**
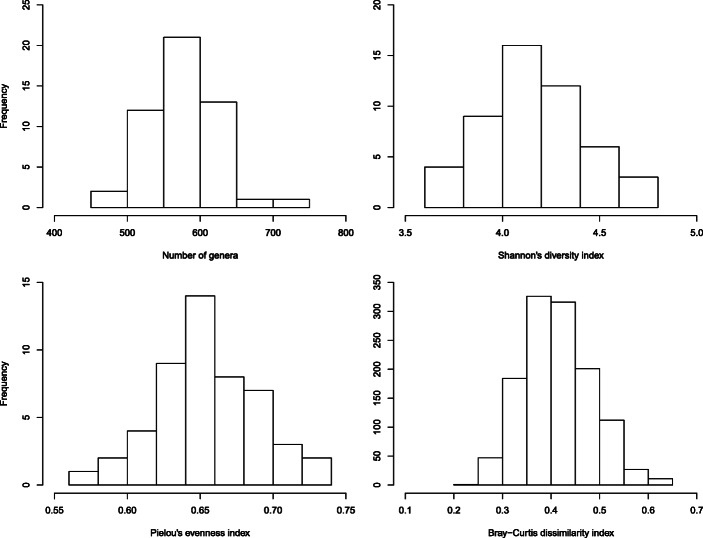
Bacterial richness, diversity, evenness, and dissimilarity indices in 50 schools

The median number of bacterial genera identified in the 50 schools was 577 (range, 470-–705) (Fig. 2). The Shannon-Weaver diversity index ranged from 3.61 to 4.72 (median, 4.14) and the Pielou’s evenness index from 0.57 to 0.72 (0.65). The median of the Bray-Curtis dissimilarity index (1225 unique pairs of schools) was 0.41 (range, 0.23 to 0.63), indicating that one-half of paired schools were at least 40% dissimilar in their genus composition. For the 499 classrooms, the Bray-Curtis index (more than 124,000 pairs) ranged from 0.08 to 0.99 (median, 0.66).

### Relative abundance of dominant genera and hierarchical clustering of schools

**Fig. 3:**
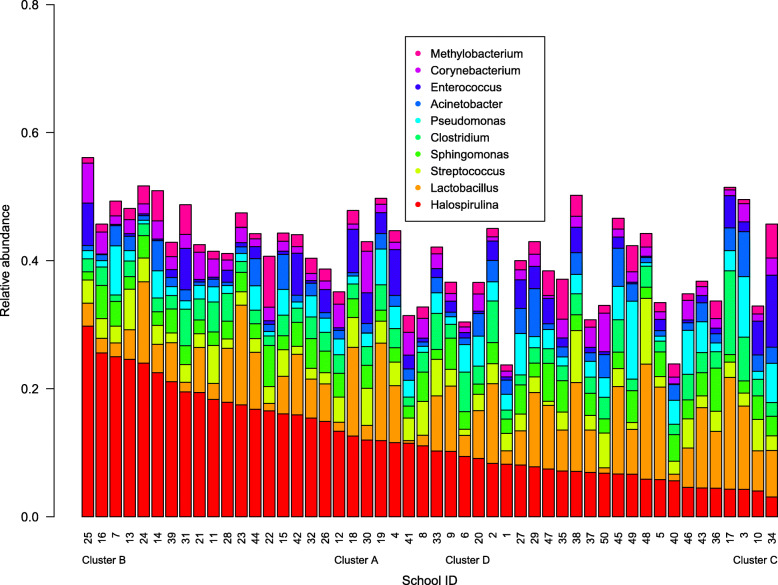
Relative abundance of the top ten bacterial genera within each school and four clusters of schools by hierarchical clustering. Numbers (school IDs) were highlighted with green color for the cluster A, red for the cluster B, yellow for the cluster C, and orange for the cluster D

We examined relative abundance of the top ten most abundant genera within each school (Fig. 3) and the top 30 genera for all 50 schools (Supplemental Figure 2). In 32 of 50 schools (64%), cumulative relative abundance of the top ten genera was 0.4 or higher (Fig. 3). The cumulative relative abundance of the genera *Halospirulina* and *Lactobacillus* was higher than any other genus for all schools, except for school number 34 where *Enterococcus* was more abundant than the summation of the two. The genus *Pseudomonas* was most abundant as a single genus in schools 46 and 49. Figure 3 also presents four clusters created by hierarchical clustering of 50 schools and Supplemental Figure 3 shows that each of the clusters had characteristic genus composition. The cluster A included schools with *Halospirulina* at a medium level in relative abundance (~ 0.1 within the cluster) and *Bacillus* (0.075) along with low abundance of *Lactobacillus* (< 0.02) (Supplemental Figure 3). The cluster B included schools with the highest within-school relative abundance of *Halospirulina* (~ 0.2). The cluster C was composed of schools with lower relative abundance (0.06) of *Halospirulina* along with medium abundance of *Lactobacillus* (~ 0.07) and higher relative abundances of *Sphingomonas* and *Pseudomonas* than other clusters. The cluster D consisted of schools with higher relative abundance of *Lactobacillus* (0.12) than those in other clusters. In the clusters A and C, the cumulative relative abundance of the top 10 genera was generally lower than the clusters B and D. When average water damage scores were compared among the clusters, cluster A had a significantly lower score than cluster D. Cluster D had the highest mean score of all the clusters (score of the cluster D > C > B > A). Multiple comparisons adjusted with Tukey’s HSD showed that all pairwise comparisons were significantly different, except two pairs of clusters (A and B, and C and D) that were not different and the clusters A and C that were marginally different (Supplemental Figure 4).

### Association of richness and community composition with school/classroom characteristics

**Fig. 4:**
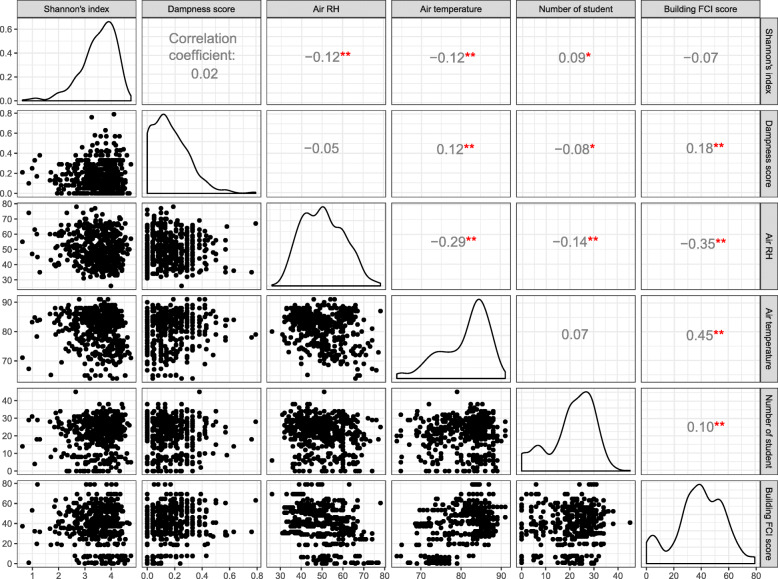
Correlations among bacterial diversity index and environmental parameters. The values on the upper diagonal of the correlation matrix are Pearson correlation coefficients with the significance level for the bivariate scatter plots on the lower diagonal. The distribution of each variable is shown on the diagonal. FCI, facility condition assessment index; *P* values: **P value ≤ 0.05, *0.05 < *P* value ≤ 0.1

Distributions of continuous environmental variables and their correlation coefficients with the Shannon-Weaver index are presented in Fig. 4. Shannon-Weaver index was not associated with the average water damage score and the FCI score; however, it was positively but weakly correlated with the number of students in the classroom (correlation coefficient = 0.09, *P* value = 0.06). It was also negatively but weakly associated with air RH (− 0.12, 0.01) and temperature (− 0.12, < 0.01). The FCI scores were negatively correlated with air RH (− 0.35, *P* < 0.001) and positively with air temperature (0.45, *P* < 0.001). In the rarefied genus accumulation curves (Supplemental Figure 5), the steepest slope of the initial accumulation curve and the highest plateau for the schools in the southwestern area within the city indicated the highest proportion of relatively abundant genera and the highest richness, respectively. The number of students in the classroom did not influence bacterial richness in classroom floor dust. School groups by quartile (Q1 through Q4) of classroom average water damage score showed slight differences in richness (height of plateau), and the most water-damaged schools (Q4) had a higher proportion of relatively abundant genera compared to other quartiles. The continuous increase of rarefaction curves for clusters B and D indicated the presence of many rare genera in these clusters (Supplemental Figure 6).

**Fig. 5:**
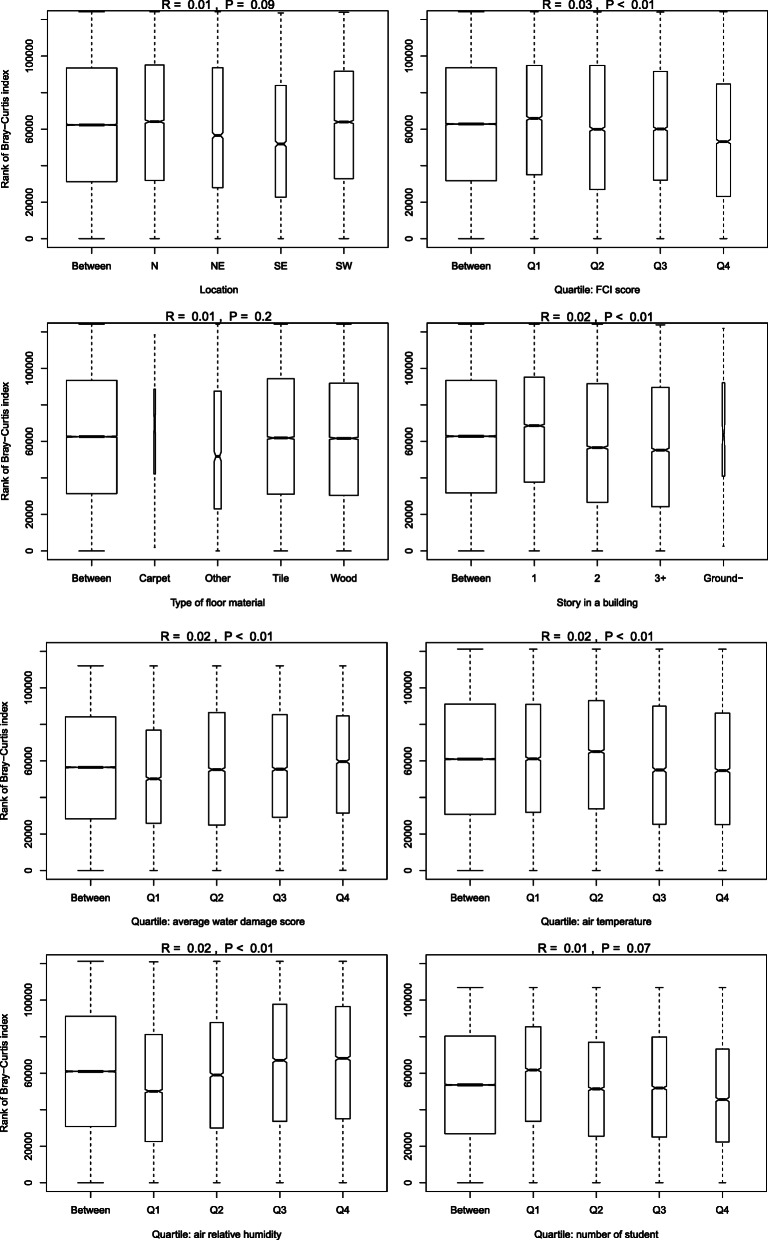
Analysis of similarity using rank of Bray-Curtis dissimilarity index over 499 samples by the group or level of categorical environmental variables

ANOSIM (Fig. 5) results indicated that the effects of the categorical variables on community composition were small (*R* values < 0.03) but significant, except for the type of floor materials. The classrooms in schools in need of least repair (Q1 of FCI score) were more dissimilar in composition than those in need of more repair (the group needing most repair was least dissimilar). The physical condition of the building (FCI score) affected dissimilarity the most among the environmental variables. Genus dissimilarity did not differ by classroom floor material type; however, it differed by floor levels of the classrooms (*R* = 0.02, *P* value < 0.01), with the first floors generally being the most dissimilar. There was a tendency that dissimilarity increased as water damage score or RH increased; whereas those with the highest temperature in air were least dissimilar. The number of students had a marginal effect with classrooms with most students (Q4) being least dissimilar. All of the full and reduced multivariate models (PERMANOVA) adjusted for other environmental variables indicated that all of the environmental factors significantly affected dissimilarity in genus composition of paired classrooms although the effect was small (Table 1), which was consistent with the results of the univariate ANOSIM analyses. Mean water damage scores of school classrooms in the southwestern or northern region of the city were significantly higher than those in the northeastern or southeastern region (Supplemental Figure 7). Because of this correlation, we constructed reduced PERMANOVA models without school area or water damage, but they yielded results similar to those of the full model. The PERMANOVA models also indicated that the effects of the physical condition of the building, area of the school, and classroom air temperature were greater than other environmental variables.

**Table 1:** Effect of environmental factors on dissimilarity in genus composition of paired classrooms using permutational multivariate analysis of variance (PERMANOVA) models

Environmental variable	Mean (SD)*	Full model (all categorical)**		Reduced model 1 (all categorical)**		Reduced model 2 (all categorical)**	
		*R*^2^	*P* value	*R*^2^	*P* value	*R*^2^	*P* value
Water damage score	0.17 (0.13)	0.01	< 0.001			0.01	< 0.001
Relative humidity	50.4 (9.9)	0.01	0.04	0.01	0.03	0.01	0.02
Temperature	81.1 (5.8)	0.01	< 0.001	0.01	< 0.001	0.02	< 0.001
Number of students	21.1 (9.3)	0.01	0.02	0.01	0.01	0.01	0.01
FCI score^†^	38.8 (17.9)	0.02	< 0.001	0.02	< 0.001	0.02	< 0.001
Area		0.02	< 0.001	0.02	< 0.001		
Floor material type		0.01	< 0.01	0.01	< 0.01	0.01	<0.01
Story in a building		0.01	< 0.001	0.01	< 0.001	0.01	< 0.001
Model residuals		0.89		0.90		0.91	

*Mean and standard deviation (SD) for the numerical variables

**All environmental variables in these models were categorical. Area: north (*n* = 159), northeast (90), southeast (90), southwest (160); type of floor material: carpet (13), tile (208), wood (223), others (55); story in a building: 1st (165), 2nd (171), 3rd, or higher (143), ground or lower (20). Continuous variable was categorized into four levels based on quartile to create a categorical variable

†*FCI* facility condition assessment index

**Fig. 6:**
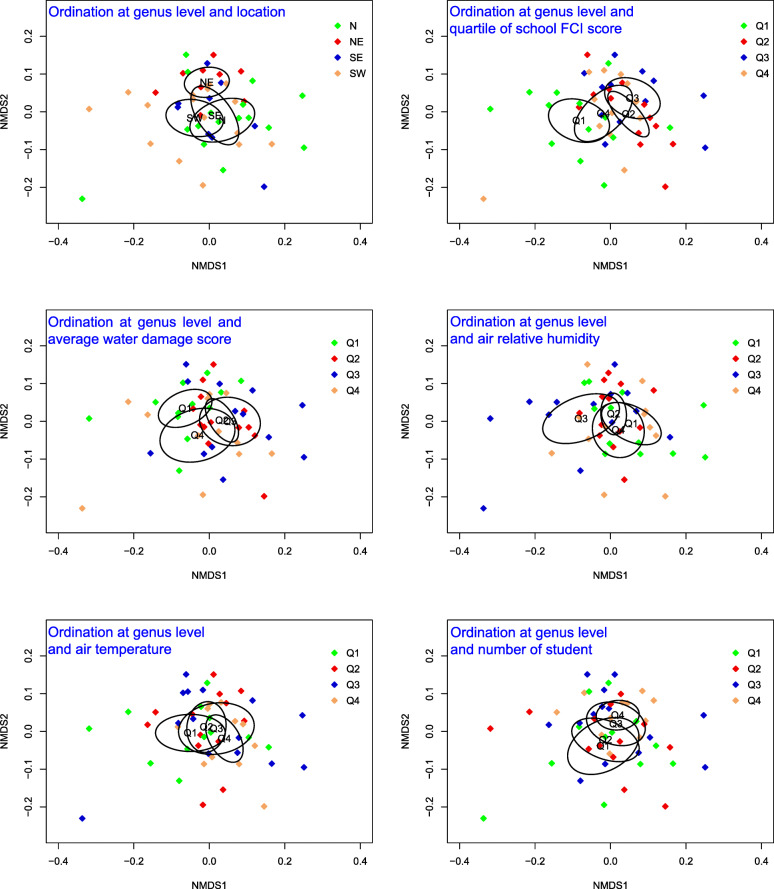
Unconstrained nonmetric multidimensional scaling (NMDS) for 50 schools (denoted by filled diamonds) grouped into quartiles (by different colors) in each environmental variable. Ellipses were constructed with a 95% confidence interval using standard error in chi-square distribution with two degrees of freedom

The first two of the three dimensions in unconstrained NMDS is presented in Fig. 6. Stress values (0.1) indicate three-dimensional ordination is fair. Schools in the northeastern area (furthest away from the center city in south) of the city had a distinct community composition compared to those in other areas, especially the southwestern and northern areas (i.e., the 95% CI ellipse of spatial ordination for schools in the northeastern area did not overlap with those of the southwestern and northern areas as shown in Fig. 6). Distance-decay model also indicated that dissimilarity in the pairwise schools slightly increased (*P* value < 0.05) with distance between the schools. The 95% CI ellipse for schools in need of the least repair (Q1) did not overlap with those for schools in Q2 and Q3 in FCI scores, which showed a different genus composition for schools in Q1. The 95% CI ellipse for schools with the least water damage (Q1) overlapped only slightly with those for schools in Q2 and Q3, indicating a different community composition for schools in Q1. However, quartile groups of RH, temperature, and the number of students did not show characteristic community composition in NMDS.

## Discussion

### Bacterial community in the school classrooms

In floor dust from 499 classrooms in 50 elementary schools in a large US city, we found that the *Proteobacteria* was the richest of all 29 phyla although the *Firmicutes* was most abundant. Our top three most abundant phyla (*Firmicutes*, *Proteobacteria*, and *Actinobacteria*) in the classrooms were consistent with those of a longitudinal study of outdoor microbiomes in the atmosphere near surface in two Colorado cities [37]. In our school classrooms, human skin-associated (*Lactobacillus*, *Streptococcus*, *Corynebacterium*, and *Acinetobacter*) and human and animal feces-associated genera (*Enterococci*) [28, 38, 39] had rich species (155 in total) but were not predominant in relative abundance. In contrast, we found *Halospirulina* and *Pseudomonas* in great abundance, possibly carried on occupants’ shoes into the classrooms as a soil component, and *Sphingomonas* and some *Acinetobacter* spp., possibly introduced from outdoor air after being released from plant leaf surfaces [37, 40]. These outdoor bacteria were not rich in species but relatively abundant in our classroom floor dust. The genus *Clostridium*, the 5th most abundant genus in our study, is ubiquitous in environments such as soils, sediments of a body of water and rivers, sewage, and human and animal intestinal tracks [41]. *Methylobacterium*, the 10th most abundant genus in our study, is also ubiquitous in nature and one of the common outdoor airborne bacteria, but generally found as part of a transient flora or as accidental contaminants [42]. In summary, human-associated bacterial genera in the top ten were more diverse (155 species) but less abundant (relative abundance = 0.16) than outdoor environment-associated genera that were much less diverse (54 species) but more abundant (0.23) in our classroom dust. This finding may indicate that classroom environments had more proliferation of non-human-associated bacteria than those originated from humans, although human occupants were one of the main sources for classroom microbiome.

Grice et al. reported that 99% of the human skin microbiome was represented by four phyla: *Actinobacteria*, *Firmicutes*, *Proteobacteria*, and *Bacteroidetes* [43]. Of these, 62% were placed in three genera: *Corynebacteria* (the phylum *Actinobacteria*), *Propionibacteria* (*Actinobacteria*), and *Staphylococci* (*Firmicutes*). A Finnish study of four urban homes estimated that 16 to 41% of sequences (relative abundance) identified in house floor dust might have originated from occupant’s skin and another 6 to 40% likely from non-skin body parts of humans [18]. Our finding that human-associated bacteria in classroom dust were diverse but not relatively abundant was a contrast to the findings of studies on house dust. We also found that gram-negative bacteria were more abundant and richer than gram-positive bacteria. A literature search indicated that there was one study reporting abundant gram-negative bacteria in the air of university classrooms [44] while numerous studies reported abundant gram-positive bacteria in homes and other indoor environments. A study of four homes in Finland [18] reported a predominance of gram-positive bacteria (59% of species-level OTUs and 79% in relative abundance) in house dust while we found a lower proportion (47% of genus-level OTUs and 46% in relative abundance) in classroom dust. A study of two nursing homes in Finland also reported dominantly abundant gram-positive bacteria in dust samples [45]. A literature review also summarized that the bacterial community in house dust is dominated by gram-positive bacteria [46] However, we are not aware of any study reporting predominance of gram-negative bacteria in floor dust of elementary school classrooms. Gram-negative bacteria generally require higher water activity for growth than gram-positive bacteria and fungi, and outdoor air is a rich source for gram-negative bacteria such as the phyla *Proteobacteria* and *Bacteroidetes* [46, 47]. Our findings indicated that the classroom environments in our study might have been frequently damp, and that outdoor sources might have played a more important role in shaping the microbiome in classroom floor dust than human sources. These also imply that there might be a characteristic difference in bacterial community and abundance between surface dust in residential buildings and floor dust in school classrooms.

In our study, we unexpectedly found that *Halospirulina* spp. were the most abundant in many schools, especially in the 18 schools categorized into the cluster B by hierarchical clustering. *Cyanobacteria* are photosynthetic prokaryotes that comprise approximately 165 genera and 1500 species and produce cyanotoxins in some species [48]. Their habitats cover a wide range of environments including water with low or high salt concentration, terrestrial, and subaerial. Moreover, they have remarkable survivability in extreme temperatures (hot springs, Arctic and Antarctic lakes, snow, and ice), and in dried ponds with high saline concentrations [49–51]. An office building study in Kuala Lumpur, Malaysia, identified *Cyanobacteria* in indoor air using the culture method that were likely tracked in from outdoor soils by humans or aerosolized from the soil of the indoor potted plants [40]. However, *Halospirulina* species were not cultured from their study. There are few published studies on the genus *Halospirulina*, but we are not aware of any literature that documents the presence of this particular genus in classroom environments. Overall, *Cyanobacteria* are usually abundant in outdoor air of hot and humid tropical regions, favor high water content for growth, and have exceptional survivability in extreme conditions [49, 52]. We postulate that after introduction from outdoors into the buildings, *Halospirulina* might have proliferated in damp conditions of their microenvironments during the wet/dry cycles from recurring water damage. In addition, their extraordinary survivability in any extreme conditions of the microenvironments might have resulted in their abundance in these schools.

### Effect of environments on richness, abundance, and community composition

In the rarefied genus accumulation curves, the classrooms in schools in the southwestern area of the city where there was a higher degree of water damage, or in the school buildings requiring the least repair (lower FCI score) had a high proportion of relatively abundant genera and an increased bacterial richness within the group [53]. Our finding of the highest richness in bacterial genera in the classrooms with the most water damage compared with other quartile groups was similar to the finding from a study of 198 homes in the southern New England region of the USA. They reported an association between water leaks and increased bacterial richness in living room surface dust [16]. Classrooms with low air RH (Q1) had the fewest abundant genera and many rare genera compared to others with higher air RH. Low RH in indoor air may decrease the available water for bacterial growth on the substrates or in microenvironments, which might have prevented the proliferation of microbes and resulted in the fewest abundant genera [46]. The classrooms in schools requiring less repair or relatively lower temperature tended to have higher RH in our study. Taken together, our study may indicate that the location of school affects bacterial richness and that a high degree of water damage and high humidity also increases the bacterial richness in floor dust in school classrooms.

Bacterial composition and abundance in built environments might depend on environmental factors such as ventilation type, local climate, type of indoor fomites, season, and geographical area [45, 54, 55]. A study of living room floor dust from 286 homes in Germany [56] found that the only significant environmental factor affecting bacterial community composition was natural ventilation in winter (but not in summer). We found that the effects of most environmental factors on the bacterial community composition in classroom dust were small but statistically significant. A New England home study in the USA also found subtle (*R* ≤ 0.06 in ANOSIM) but significant effects of home type (single vs. multifamily), home location (urban vs. suburban), and presence of pets on community composition [16]. Our finding of the effect of water damage on community composition in classroom dust is comparable to that in a Finnish microbiome study of water-damaged homes with and without renovation [57]. We also observed a higher similarity in community composition among the classrooms with a higher number of students. Dannemiller et al. also found that higher occupancy was associated with lower compositional variation in living room surface dust [16]. Additionally, we found that classrooms on the first floor showed higher dissimilarity within the group compared to those on other floors, possibly indicating a greater effect of rich outdoor bacteria tracked into first-floor classrooms than onto other floors. Our multivariate models adjusted for other environmental factors indicated a significant effect of floor material on composition. Perhaps, the classrooms with carpeted floors might have a more dissimilar composition compared to those with smooth floors (Fig. 5). In aggregate, these findings implicate that individual environmental factors might marginally influence bacterial community composition in classroom floor dust without a dominant single factor. This could indicate resilience of the indoor microbiome once it is established, unless there is a dramatic change in the environment such as water intrusion.

A strength of our unique school study is that we had a large sample size (from 499 classrooms, 7.6 million sequences) from 50 schools, which allowed us to reliably examine bacterial diversity and community composition of classroom floor dust and determine the effect of environmental factors within the study area. Yet, our cross-sectional study was conducted in summertime only, a limitation that may influence generalizing the results. The indoor microbiome is influenced by various outdoor sources that can vary by season [37, 45, 56]. However, because we collected cumulative dust that was likely to be settled or tracked in over extended periods of time (perhaps multiple seasons), the microbiomes in our dust might not be substantially affected by seasonal changes. Primer biases and factors such as gene copy number could result in differential amplification and identification of certain taxa [58]. We also recognize that extraction bias might have potentially influenced our identification [20].

## Conclusions

From our cross-sectional study of 50 elementary schools, we found that outdoor bacterial sources and numerous indoor environmental conditions might have collectively played important roles in shaping classroom microbiome in floor dust, while human occupants remained as one of the important sources. In addition, school or classroom environmental factors significantly affected bacterial richness and community composition although their effects were subtle, indicating the relative stability of indoor microbiomes to environmental stress once established. Our findings demonstrate that microbiomes in school classrooms might be different from those in homes, which suggests that the health implication of exposure to microbiomes in schools could be different from that in residential environments. Thus, epidemiologic and clinical studies are warranted to better understand the effect of school or classroom microbiomes on health in school staff and students. The characteristics of bacterial microbiomes we found in this study will guide our future epidemiologic analysis of schoolteachers’ health related to microbial exposures.

## Supplementary Information

**Additional file 1: Supplemental Figure 1.** Relative abundance of all 29 bacterial phyla identified in 499 samples. **Supplemental Figure 2.** Relative abundance of the most abundant top 30 genera. **Supplemental Figure 3.** The most abundant top 30 genera for each of the four clusters of dendrogram. **Supplemental Figure 4.** Box plot of average water damage score for each of the four clusters of dendrogram. Each box plot shows [median-1.5 × (interquartile range, IQR)], 25^th^ percentile, median (thicker horizontal line within the box), 75^th^ percentile, and [median + 1.5 × (IQR)]. IQR is defined as (75^th^ percentile)-(25^th^ percentile). Values outside the whiskers are considered outliers and denoted as an open circle. **Supplemental Figure 5.** Each panel presents rarefied genus accumulation curves by the level of each environmental variable. The second plot on the top row shows only six schools in the top three highest richness or the bottom three lowest richness. Vertical line: the number of sampled sequences normallized to compare richness; horizontal line: estimated number of genus by rarefaction at the same number of sampled DNA sequences. Overlapping labels indicate the similar level of richness. **Supplemental Figure 6.** Rarefied genus accumulation curves for the four clusters of dendrogram. **Supplemental Figure 7.** Distribution of average damage score for the four groups of schools by location. Red dot denotes mean value. Each box plot shows [median-1.5 × (interquartile range, IQR)], 25^th^ percentile, median (thicker horizontal line within the box), 75^th^ percentile, and [median + 1.5 × (IQR)]. IQR was defined as (75^th^ percentile)-(25^th^ percentile). Dots outside the whiskers are considered outliers.

## Data Availability

The datasets generated and/or analyzed during the current study are available in the NCBIs sequence read archive database (https://www.ncbi.nlm.nih.gov/sra) and the NCBI BioSample database repository (https://www.ncbi.nlm.nih.gov/biosample). The BioProject ID is PRJNA661085.

## Abbreviations

OTU: Operational taxonomic unit
RH: Relative humidity
FCI: Facility condition index
Tukey HSD: Tukey honestly significant difference test
ANOSIM: Analysis of similarity
PERMANOVA: Permutational multivariate analysis of variance
NMDS: Nonmetric multidimensional scaling
