# Correction to: Bacterial community assemblages in classroom floor dust of 50 public schools in a large city: characterization using 16S rRNA sequences and associations with environmental factors

**DOI:** 10.1186/s40168-021-01005-0

**Published:** 2021-02-12

**Authors:** Ju-Hyeong Park, Angela R. Lemons, Jerry Roseman, Brett J. Green, Jean M. Cox-Ganser

**Affiliations:** 1 Respiratory Health Division, National Institute for Occupational Safety and Health, Morgantown, WV USA; 2 Health Effect Laboratory Division, National Institute for Occupational Safety and Health, Morgantown, WV USA; 3 Philadelphia Federation of Teachers Health & Welfare Fund & Union, Philadelphia, PA USA

**Correction to: Microbiome (2021) 9:15**

**https://doi.org/10.1186/s40168-020-00954-2**

Following publication of the original article [1], the authors identified an error in Fig. 3. The color highlights on the school IDs (X-axis tick label) and the cluster name for the four clusters were removed.

The correct figure is provided below.

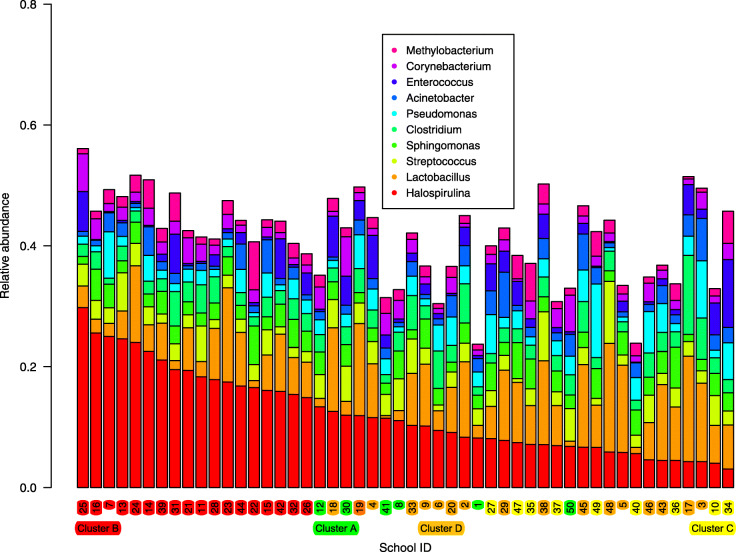



The original article [1] has been corrected.
