# Supplementary material for: Bacterial community assemblages in classroom floor dust of 50 public schools in a large city: characterization using 16S rRNA sequences and associations with environmental factors
Source: Microbiome. 2021 Jan 20;9:15. doi: 10.1186/s40168-020-00954-2 (PMC7819239; doi:10.1186/s40168-020-00954-2)
Supplement: Supplementary file 2 — Additional file 1: Supplemental Figure 1. Relative abundance of all 29 bacterial phyla identified in 499 samples. Supplemental Figure 2. Relative abundance of the most abundant top 30 genera. Supplemental Figure 3. The most abundant top 30 genera for each of the four clusters of dendrogram. Supplemental Figure 4. Box plot of average water damage score for each of the four clusters of dendrogram. Each box plot shows [median-1.5 × (interquartile range, IQR)], 25th percentile, median (thicker horizontal line within the box), 75th percentile, and [median + 1.5 × (IQR)]. IQR is defined as (75th percentile)-(25th percentile). Values outside the whiskers are considered outliers and denoted as an open circle. Supplemental Figure 5. Each panel presents rarefied genus accumulation curves by the level of each environmental variable. The second plot on the top row shows only six schools in the top three highest richness or the bottom three lowest richness. Vertical line: the number of sampled sequences normallized to compare richness; horizontal line: estimated number of genus by rarefaction at the same number of sampled DNA sequences. Overlapping labels indicate the similar level of richness. Supplemental Figure 6. Rarefied genus accumulation curves for the four clusters of dendrogram. Supplemental Figure 7. Distribution of average damage score for the four groups of schools by location. Red dot denotes mean value. Each box plot shows [median-1.5 × (interquartile range, IQR)], 25th percentile, median (thicker horizontal line within the box), 75th percentile, and [median + 1.5 × (IQR)]. IQR was defined as (75th percentile)-(25th percentile). Dots outside the whiskers are considered outliers. [file 40168_2020_954_MOESM2_ESM.docx]

Bacterial community assemblages in classroom floor dust of 50 public schools in a large city: characterization using 16s rRNA sequences and associations with environmental factors

Ju-Hyeong Park^1,*^, Angela Lemons^1^, Jerry Roseman^2^, Brett Green^1^, Jean Cox-Ganser^1^

1 Respiratory Health Division, National Institute for Occupational Safety and Health, Morgantown, WV, USA

2 Philadelphia Federation of Teachers Health & Welfare Fund & Union, Philadelphia, PA, USA

* Corresponding author: Ju-Hyeong Park

Email: [gzp8@cdc.gov](mailto:gzp8@cdc.gov); mailing address: Field Studies Branch, Respiratory Health Division, National Institute for Occupational Safety and Health, Centers for Disease Control and Prevention, 1095 Willowdale Road, Morgantown, WV 26505.


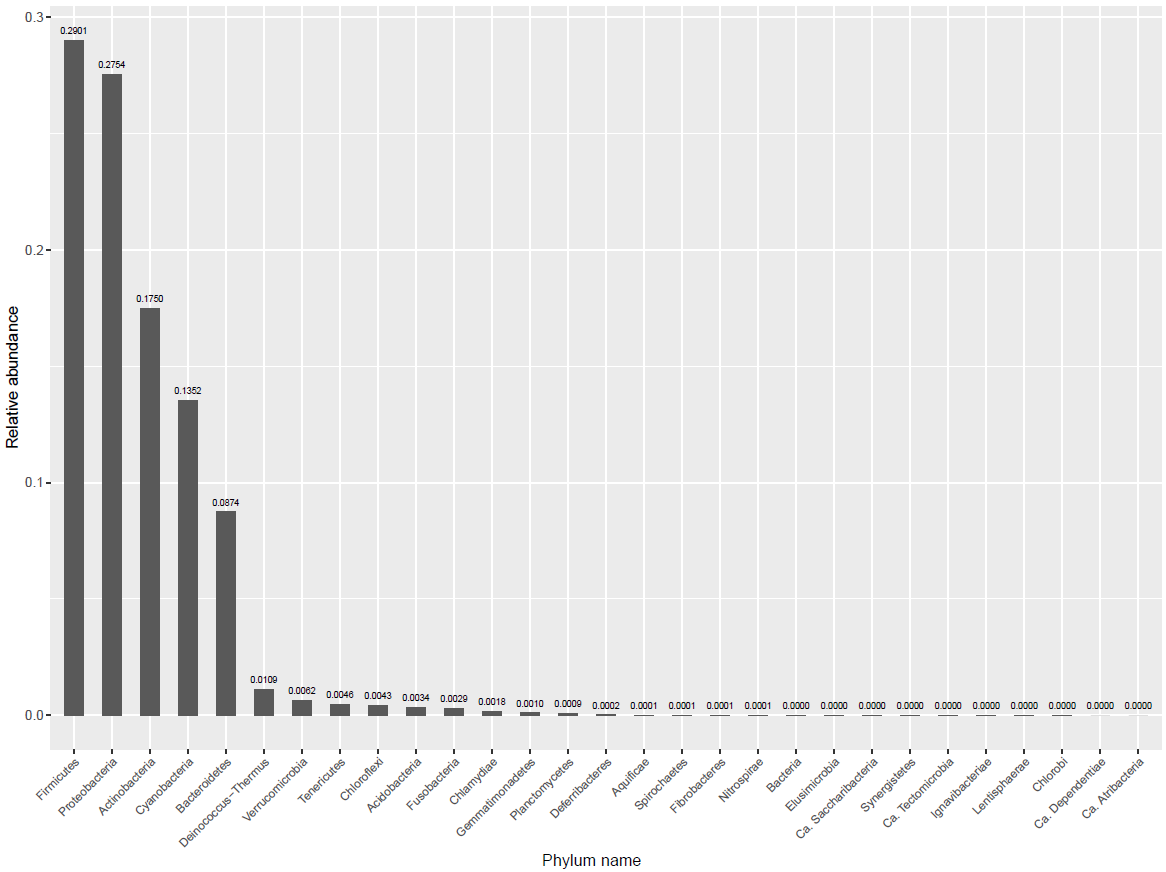


Supplemental Figure 1. Relative abundance of all 29 bacterial phyla identified in 499 samples.


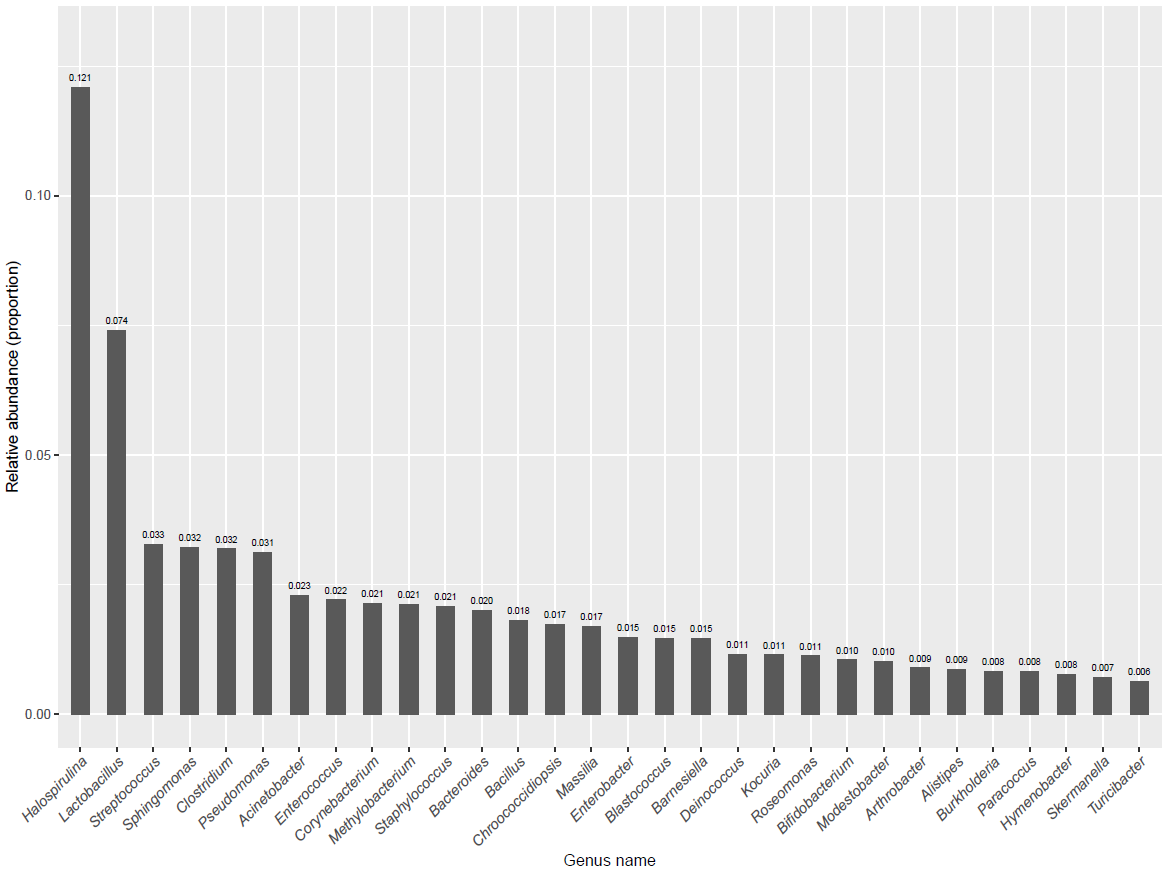


Supplemental Figure 2. Relative abundance of the most abundant top 30 genera.


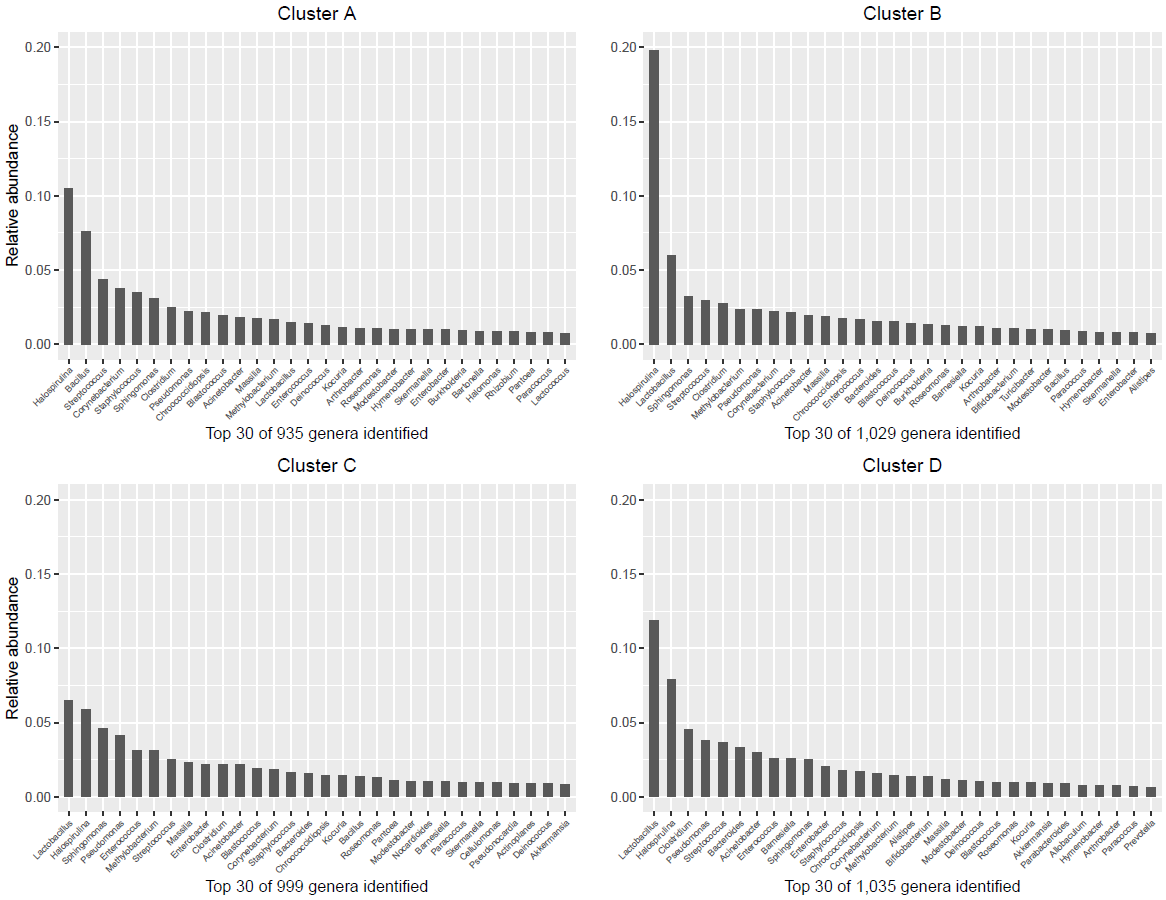


Supplemental Figure 3. The most abundant top 30 genera for each of the four clusters of dendrogram.


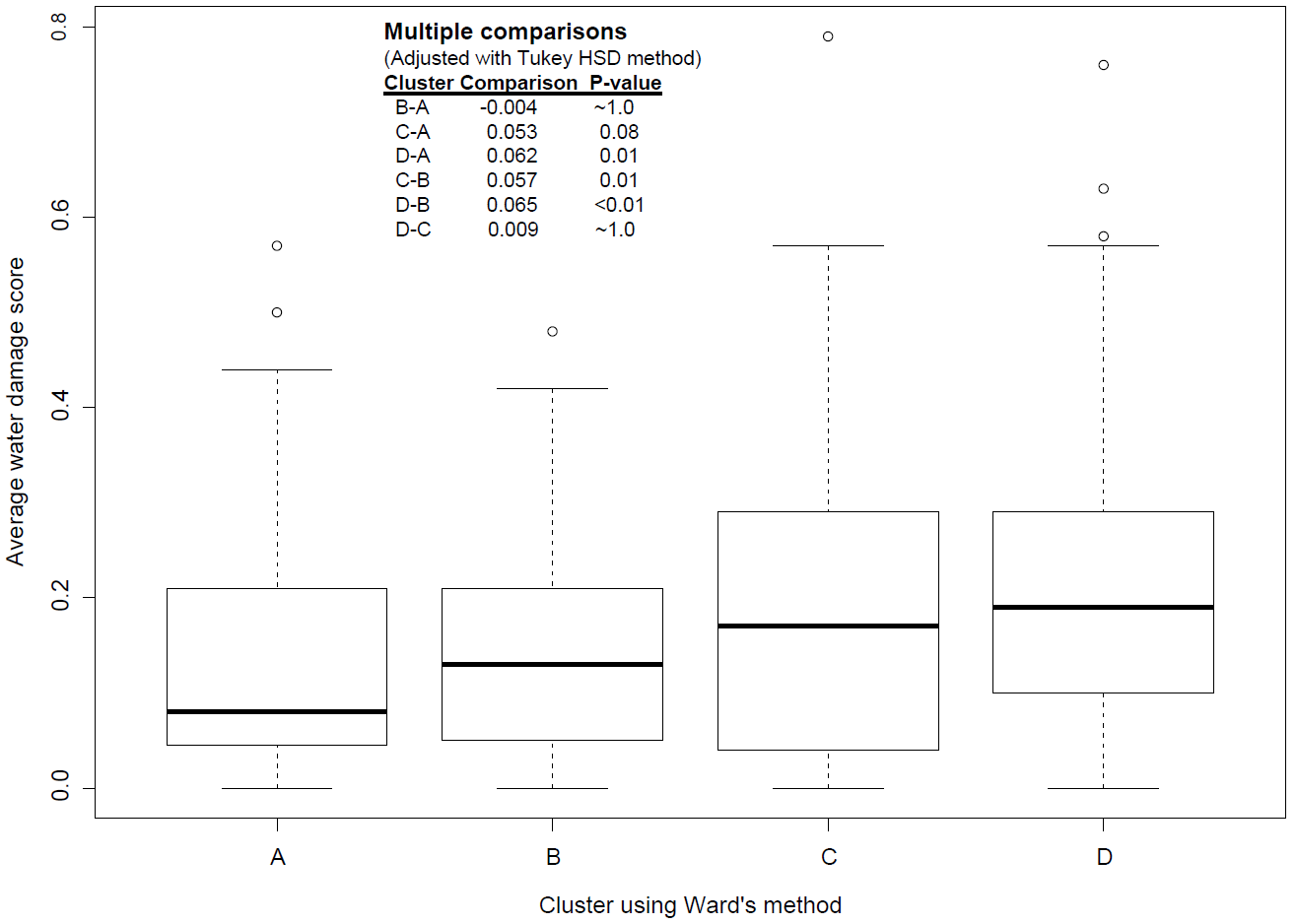


Supplemental Figure 4. Box plot of average water damage score for each of the four clusters of dendrogram. Each box plot shows [median-1.5×(interquartile range, IQR)], 25^th^ percentile, median (thicker horizontal line within the box), 75^th^ percentile, and [median+1.5×(IQR)]. IQR is defined as (75^th^ percentile)-(25^th^ percentile). Values outside the whiskers are considered outliers and denoted as an open circle.


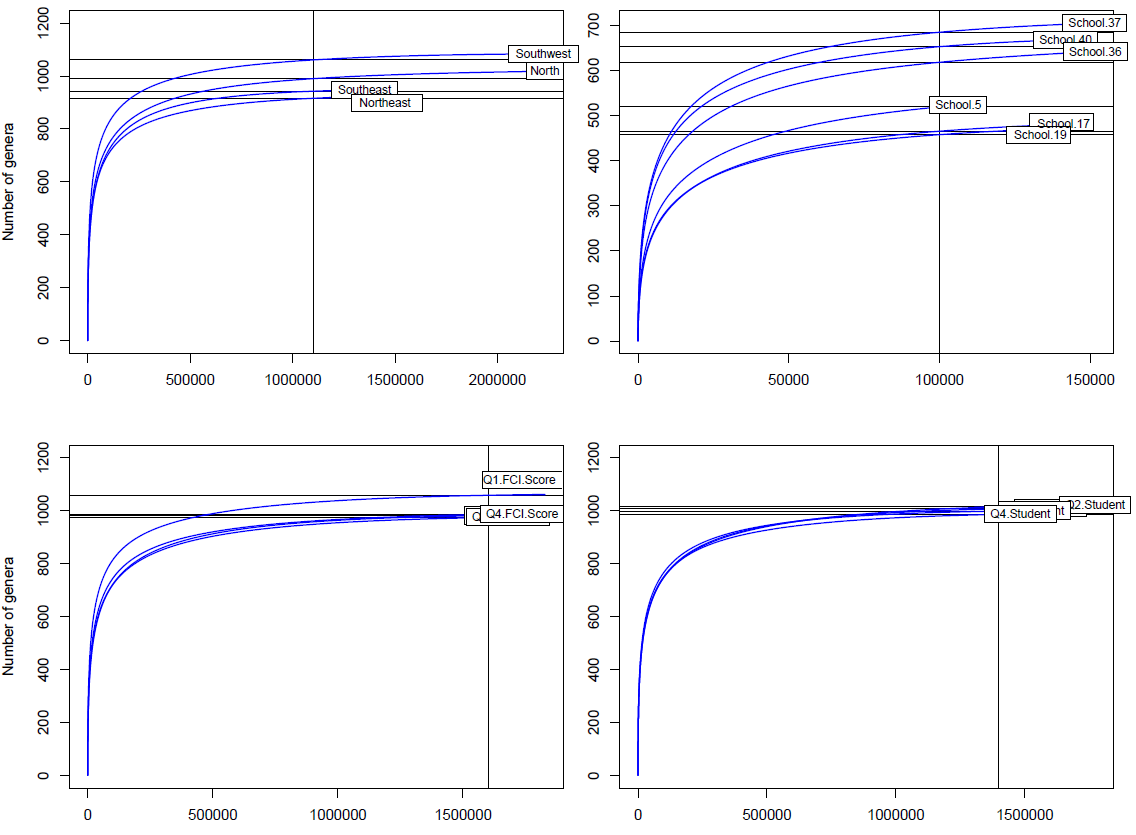


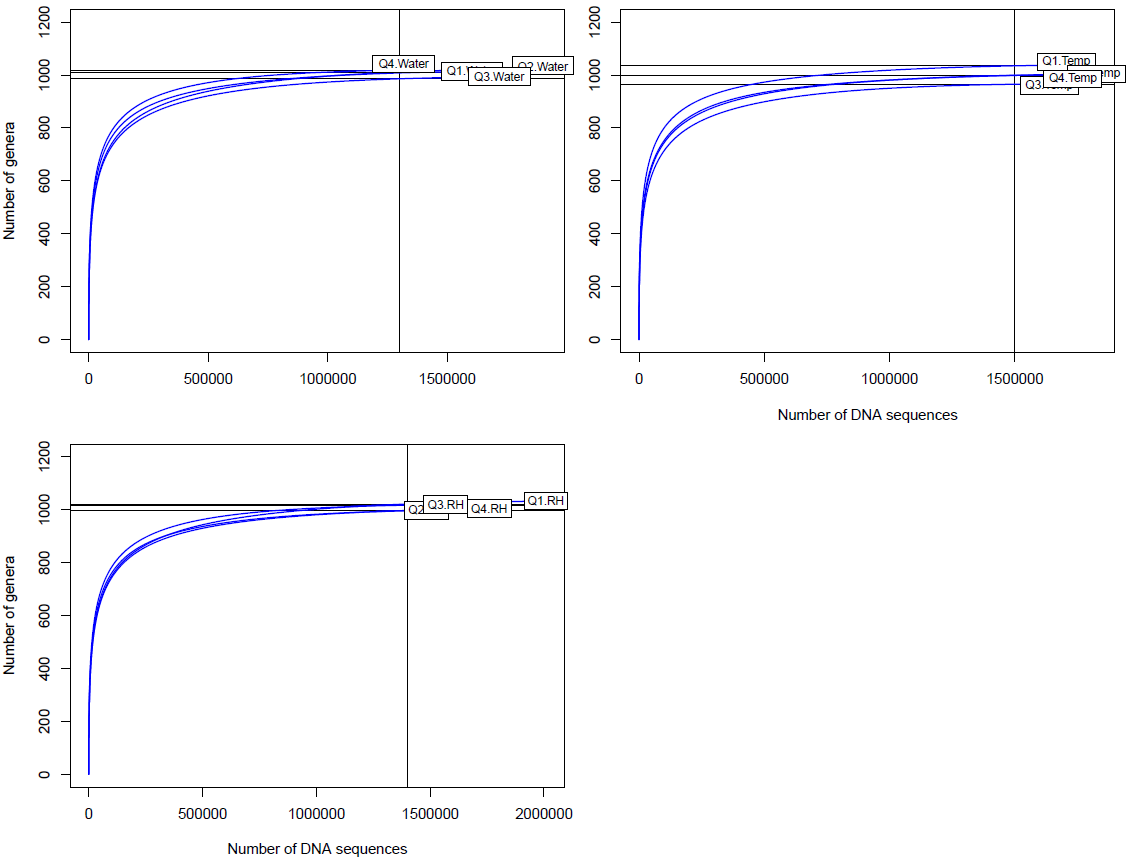


Supplemental Figure 5. Each panel presents rarefied genus accumulation curves by the level of each environmental variable. The second plot on the top row shows only six schools in the top three highest richness or the bottom three lowest richness. Vertical line: the number of sampled sequences normallized to compare richness; horizontal line: estimated number of genus by rarefaction at the same number of sampled DNA sequences. Overlapping labels indicate the similar level of richness.


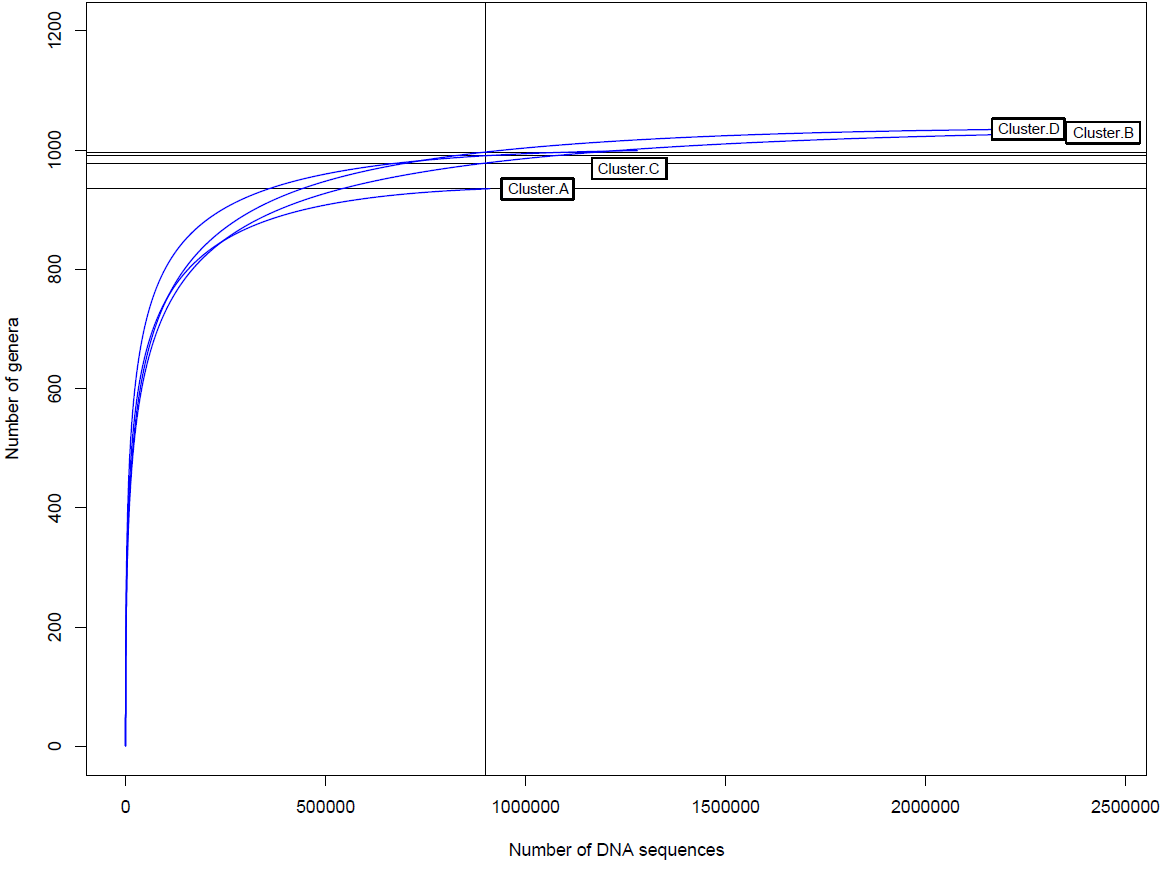


Supplemental Figure 6. Rarefied genus accumulation curves for the four clusters of dendrogram.


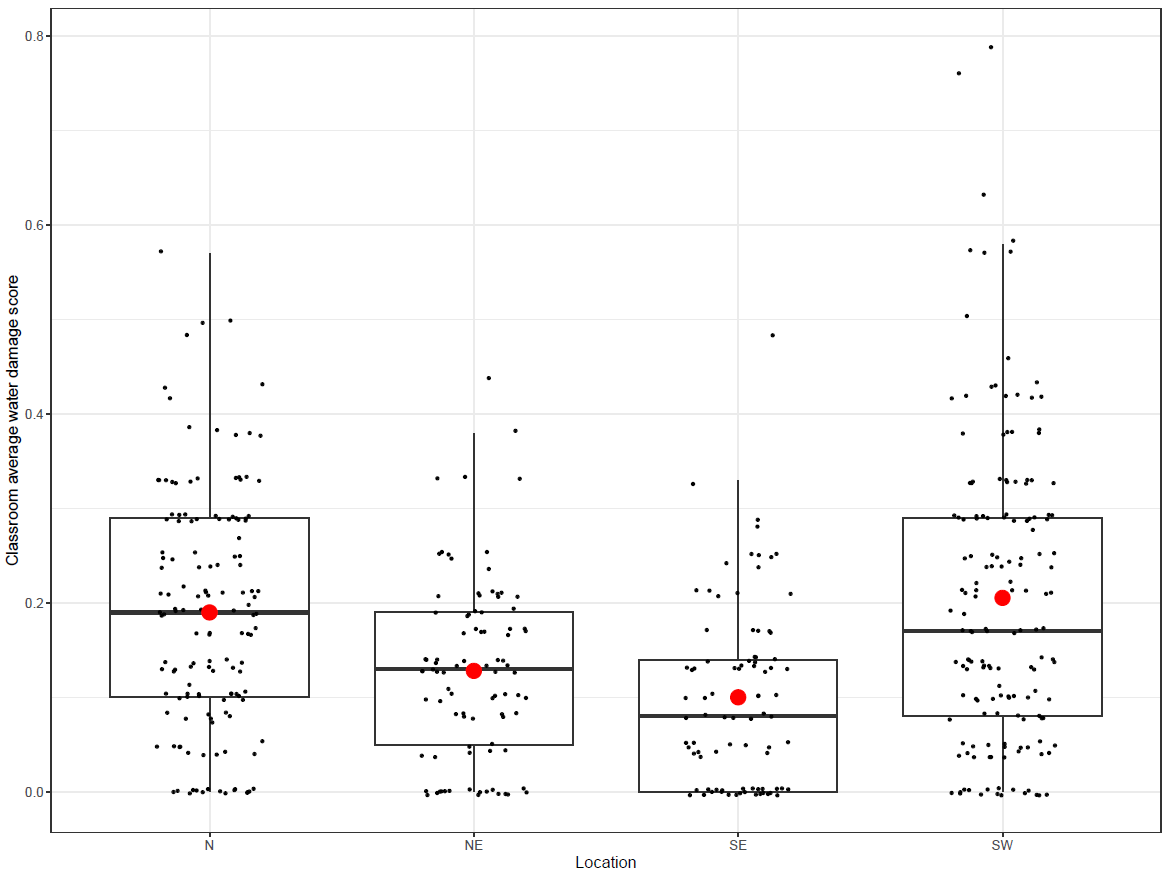


Supplemental Figure 7. Distribution of average damage score for the four groups of schools by location. Red dot denotes mean value. Each box plot shows [median-1.5×(interquartile range, IQR)], 25^th^ percentile, median (thicker horizontal line within the box), 75^th^ percentile, and [median+1.5×(IQR)]. IQR was defined as (75^th^ percentile)-(25^th^ percentile). Dots outside the whiskers are considered outliers.
